# Triangle network motifs predict complexes by complementing high-error interactomes with structural information

**DOI:** 10.1186/1471-2105-10-196

**Published:** 2009-06-27

**Authors:** Bill Andreopoulos, Christof Winter, Dirk Labudde, Michael Schroeder

**Affiliations:** 1 Biotechnology Center (BIOTEC), Technische Universität Dresden, 01307 Dresden, Germany; 2 nanometis, Tatzberg 47-49, 01307 Dresden, Germany

## Abstract

**Background:**

A lot of high-throughput studies produce protein-protein interaction networks (PPINs) with many errors and missing information. Even for genome-wide approaches, there is often a low overlap between PPINs produced by different studies. Second-level neighbors separated by two protein-protein interactions (PPIs) were previously used for predicting protein function and finding complexes in high-error PPINs. We retrieve second level neighbors in PPINs, and complement these with structural domain-domain interactions (SDDIs) representing binding evidence on proteins, forming PPI-SDDI-PPI triangles.

**Results:**

We find low overlap between PPINs, SDDIs and known complexes, all well below 10%. We evaluate the overlap of PPI-SDDI-PPI triangles with known complexes from Munich Information center for Protein Sequences (MIPS). PPI-SDDI-PPI triangles have ~20 times higher overlap with MIPS complexes than using second-level neighbors in PPINs without SDDIs. The biological interpretation for triangles is that a SDDI causes two proteins to be observed with common interaction partners in high-throughput experiments. The relatively few SDDIs overlapping with PPINs are part of highly connected SDDI components, and are more likely to be detected in experimental studies. We demonstrate the utility of PPI-SDDI-PPI triangles by reconstructing myosin-actin processes in the nucleus, cytoplasm, and cytoskeleton, which were not obvious in the original PPIN. Using other complementary datatypes in place of SDDIs to form triangles, such as PubMed co-occurrences or threading information, results in a similar ability to find protein complexes.

**Conclusion:**

Given high-error PPINs with missing information, triangles of mixed datatypes are a promising direction for finding protein complexes. Integrating PPINs with SDDIs improves finding complexes. Structural SDDIs partially explain the high functional similarity of second-level neighbors in PPINs. We estimate that relatively little structural information would be sufficient for finding complexes involving most of the proteins and interactions in a typical PPIN.

## Background

Protein-protein interaction networks (PPINs) derived from high-throughput studies are known to have many errors [1,2]. Data from different studies usually exhibit low overlap; for instance, two large-scale human interactome screens [3,4] share only six interactions, while each has several thousand interactions [5-7]. In some PPINs, more than 50% of reported interactions are estimated to be false positives (FPs) or wrong interactions [8,9]. Moreover, current PPINs are incomplete with an estimated false negative (missing interactions) rate approaching 90% [10-12]. False positives often result when the matrix model, which fully connects the pray and bait proteins, is used for interpreting results of affinity purification followed by mass spectrometry experiments [13].

Not all interactions occur at the same place and time in all cellular states. This implies that representing a PPIN as a set of binary protein-protein interactions (PPIs) is often incomplete [14]. Instead, one wants to restructure protein complexes in PPINs, which are modular units of physical interactions occurring at the same time and cellular component [15,16]. For predicting complexes one wants to include complementary data, such as structural domain-domain interactions (SDDIs) representing binding evidence on proteins [17-22]. At the same time, one wants to leave out of predicted complexes the false positives [22-26].

It was proposed that triangle network motifs represent the basic building blocks of PPINs [27-32]. In this paper, we complement PPIs with SDDIs to form *PPI-SDDI-PPI triangle network motifs*. Triangle network motifs integrate high-throughput PPINs with complementary knowledge, such as structural data, to account for missing edges [25,33-38]. Our proposed paradigm of *PPI-SDDI-PPI triangle network motifs *integrate:

• PPINs from high-throughput experimental studies, which have considerable coverage but also errors, and

• SDDIs that are known to physically mediate PPIs and may be missing in PPINs [39-49].

**Figure 1:**
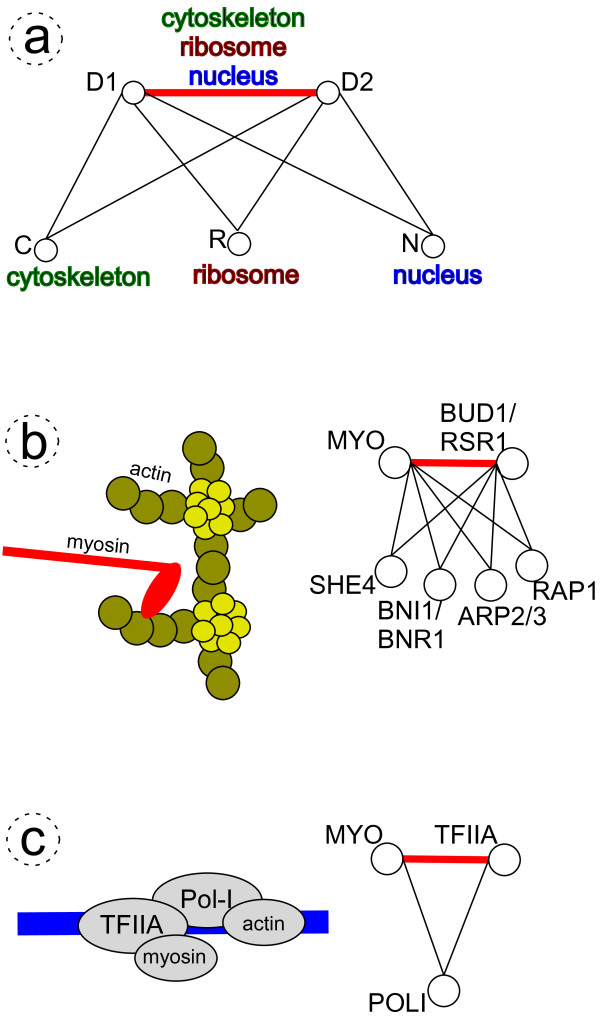
**PPIs (black) and structural SDDIs (red)**. **(a) **Theme of three PPI-SDDI-PPI triangles sharing the same SDDI. The red SDDI edge is involved in all three triangles. Proteins *D*1 and *D*2 may interact physically with *C *in the cytoskeleton, or *R *in the ribosome, or *N *in the nucleus. The transitive module hypothesis suggests two proteins such as *D*1 and *D*2 that share many common interaction partners are more likely to interact than two proteins that share few common interaction partners [9]. Some PPIs have no common Gene Ontology annotation, hinting to false positives.**(b),(c) **Biological examples of myosin-actin involvement in multiple processes/locations. Their representation as PPI-SDDI-PPI triangle network motifs and themes, as found in integrations of PPINs with SDDIs. **(b) **Myosin in actin cytoskeleton organization and formation. Myosin mediates actin remodelling and vesicular transport. **(c) **Actin and nuclear myosin I (NMI) are required for transcription by RNA polymerases (Pols) I, II, III in the eukaryotic cell nucleus. Actin is directly associated with Pol I, regardless of whether Pol I is engaged in transcription, and NMI interacts with transcription initiation factor TIFIA. TIFIA is phosphorylated. Pol I is then recruited to the DNA promoter through interaction with the phosphorylated TIFIA, which brings actin and NMI into close proximity with each other. Actin, but not NMI, remains associated with Pol I during transcription elongation [121-124].

A *theme *encompasses several PPI-SDDI-PPI triangle network motifs with one SDDI edge as their common organizational principle. Figure 1a shows a theme consisting of three PPI-SDDI-PPI triangle network motifs that share one common SDDI. To demonstrate the biological relevance of triangle network motifs and themes, Figures 1b,c show myosin-actin functions in different cellular locations: cytoskeleton organization and nuclear transcription.

The purpose of PPI-SDDI-PPI triangles is to support revealing biological insights, such as finding complexes of physical interactions occurring at the same time and location [50-55]. Besides complementing PPINs with SDDIs, we additionally form triangle network motifs with other complementary datatypes (CD), such as threading results, and PubMed protein co-occurrence data, thus expanding to other PPI-CD-PPI triangles [56-59]. The complex prediction with other CD is comparable to SDDIs; this supports that the improved complex prediction results are due to a physical relation between proteins and not just coincidence [40,60,61].

A rationale for triangles and themes is the observation that proteins with common interaction partners are likely to have common functions [62-65]. Second-level neighbors in PPINs are functionally similar, and are useful for functional prediction [66-70]. By this "Guilt by Association of Common Interaction Partners" approach, themes can be tied to specific biological phenomena and processes [71-73]. For instance, it was shown for the *E. coli *and *C. elegans *transcriptional network that subgraphs matching two types of transcriptional regulatory circuit triangle – feed-forward and bi-fan – overlap with one another and form large clusters [28,74-76]. Another rationale for triangles and themes is that PPINs are "small-world" implying neighborhood clustering, where neighbors of a given node tend to interact with one another; this results in triangle network motifs of three-node interconnection patterns [77,78]. This led to the "transitive module" hypothesis that is used for predicting missing interactions, as shown in Figure 1a, where proteins with many common interaction partners are likely to interact with one another forming triangles [9].

### Extracting triangle network motifs and themes from high-throughput interaction networks

**Figure 2:**
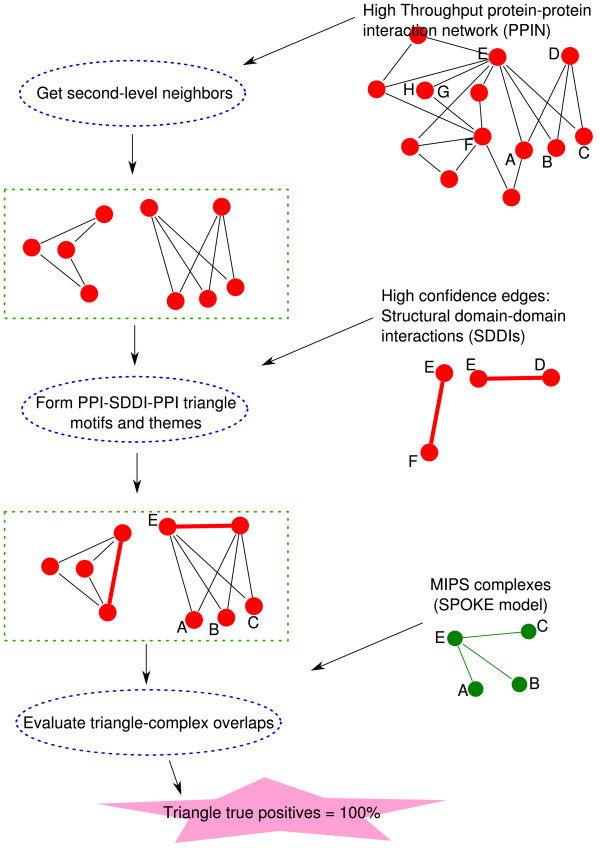
**The overall workflow of our process**. First, we extract the second-level neighbors from a PPIN. Combining these edges with a complementary data source allows finding triangles and theme motifs. Then, we compare them with known complexes such as MIPS.

Figure 2 shows the process of extracting triangle network motifs and themes. Given a high-throughput PPIN, we first extract second-level (indirect) neighbors connected by a pair of interactions. Then, we complement them with structural domain-domain interactions (SDDIs), to form PPI-SDDI-PPI triangle network motifs. In the case where a SDDI is involved in more than one triangle, we refer to it as a theme. For evaluation, we examine if the triangles' and themes' overlaps with known MIPS complexes is higher than that of the second-level (indirect) neighbors.

This paper is organized as follows. Next, we present related work on finding errors in PPINs via motifs of interconnection patterns. Then, we present the results on prediction of true positive complexes using triangles. We illustrate this with an example of myosin-actin related activities. Next, we explain the biological basis for triangles: a model for SDDIs that explains the functional similarity of second-level neighbors in PPINs. Finally, we conclude the paper with an outlook of using other data sources to complement interactomes.

### Related work

Several papers aim to find errors in PPINs by completing them for missing edges or finding false positives [79-83]. Our approach differs from all of these approaches, since we integrate structural information with PPINs derived from high-throughput studies to find triangle network motifs and themes, which can be used to predict complexes. Moreover, we offer the biological basis for the ability of this structural-PPI hybrid method to predict complexes.

A first category of work involves collecting ensembles of data, such as structural or literature information. Alber et al. (2007) [84] collect diverse high-quality data, and analyse the ensemble to produce a detailed architectural map of the nuclear pore complex. This work translates the data into spatial restraints, instead of using network motifs as in our approach. Ramirez et al. (2007) [22] assessed the quality and value of publicly available human protein network data, by comparing predicted datasets, high-throughput results from yeast two-hybrid screens, and literature-curated protein-protein interactions. This analysis revealed major differences between datasets. Rhodes et al. (2005) [85] demonstrate that a probabilistic analysis integrating model organism protein interactome data, structural domain data, genome-wide gene expression data and functional annotation data predicts nearly 40,000 interactions in humans. Bader et al. (2004) [19] perform an integrated analysis of proteomics data with data from genetics and gene expression. Combining temporal gene expression clustering with proteomics network topology provides an automated method for extracting biological subnetworks. Huang et al. (2004) [86] present POINT, the "prediction of interactome database". POINT integrates several publicly accessible databases, with emphasis placed on mouse, fruit fly, worm and yeast protein-protein interactions datasets from the Database of Interacting Proteins (DIP), followed by converting them into a predicted human interactome. POINT also incorporates correlated mRNA expression clusters obtained from cell cycle microarray databases and subcellular localization from Gene Ontology to pinpoint the likelihood of biological relevance of each predicted set of interacting proteins. Patil et al. (2005) [87] find that a combination of sequence, structure and annotation information is a good predictor of true interactions in large and noisy interactomes.

Another large body of work attempted to predict the missing interactions or assign confidences to large noisy interactomes. Some of these use network topology and others use information on SDDIs, while others use Bayesian networks or probabilistic measures. Yu et al. (2006) [68] describe predicting missing PPIs, using only the PPIN topology as observed by a high-throughput experiment. The method searches the interactome for defective cliques, nearly complete complexes of pairwise interacting proteins, and predicts the interactions that complete them. Chen et al. (2008) [88] propose using triplets of observed PPIs to predict and validate interactions. Yeast is the only data set large enough to warrant application of this method. Singhal et al. (2007) [23] present DomainGA, a computational approach that uses information about SDDIs to predict PPIs. This method achieves good prediction for the positive and negative PPIs in yeast. Pitre et al. (2006) [89] present PIPE, a system for predicting PPIs for any target pair of the yeast proteins from their primary structure. Chen et al. (2005) [24] introduce a novel measure called IRAP, "interaction reliability by alternative path", for assessing the reliability of PPIs based on the underlying PPIN topology. IRAP measure is effective for discovering reliable PPIs in large noisy PPIN datasets. Ng et al. (2003) [90] propose an integrative approach that applies SDDIs to predict and validate PPIs. Chen et al. (2005) [24] introduce a SDDI-based random forest of decision trees to infer PPIs. This method is capable of exploring all possible SDDIs and making predictions based on all the protein domains. Wu et al. (2006) [91] propose using the similarity between two Gene Ontology (GO) terms for reconstructing and predicting a yeast PPIN based solely on knowledge of functional associations between the GO annotations.

We have also experimented with using GO similarities in our approach. Chinnasamy et al. (2006) [92] present a probabilistic-based naive Bayesian network to predict PPIs using protein sequence information. This framework provides a confidence level for every predicted PPI. Jansen et al. (2003) [93] also developed an approach using Bayesian networks to predict PPIs in yeast. Han et al. (2004) [94] propose PreSPI, a domain combination based PPI prediction approach. PPIs are interpreted as the result of groups of multiple SDDIs. This approach also provides an interacting probability for PPIs. Recently, Vidal and colleagues [95] used reference sets to calculate the probability that a newly identified PPI is a true biophysical interaction, and assigned confidence scores to all PPIs in interactome networks. Yu et al. (2009) [96] assign confidence scores that reflect the reliability of each PPI, by using multiple independent sets of training positives to reduce the bias inherent in using a single training set.

Another body of work has performed large scale analysis of networks, statistical network motif analysis or error estimation, which is of interest for our work as well. Jin et al. (2007) [32] use network motifs to solve the open question about 'party hubs' and 'date hubs' which was raised by previous studies. At the level of network motifs instead of individual proteins, they found two types of hubs, motif party hubs and motif date hubs, whose network motifs display distinct characteristics on biological functions. Zhang et al. (2005) [28] observed that different types of networks exhibit different triangle profiles, providing a means for network classification. They extended the network triangle concept to an integrated network of many interaction types. Mathivanan et al. (2006) [97] analyzed the major publically available databases that contain literature curated PPI information for human proteins, finding a large difference in their content. This included BIND, DIP, HPRD, IntAct, MINT, MIPS, PDZBase and Reactome databases [98]. Chiang et al. (2007) [1] assess the error statistics in all published large-scale datasets for *S. cerevisiae*. Vidal and colleagues [99,100] used an empirically-based approach to assess the quality and coverage of existing human interactomes. They found that high-throughput human interactomes are more precise than literature-curated PPIs from publications.

Several papers used clustering or graph theoretic methods to predict complexes in PPINs. Bader et al. (2003) detected complexes as highly connected subgraphs [101]. Andreopoulos et al. (2007) detected complexes as groups of proteins with similar interaction partners [62]. Cakmak et al. (2007) [102] go beyond complexes to discover unknown pathways in organisms, using Gene Ontology (GO)-based functionalities of enzymes involved in metabolic pathways.

## Results and discussion

In our experiments, we employ three high-throughput PPINs, derived by affinity purification followed by mass spectrometry (AP/MS). Krogan06 is based on [103]. Gavin06MATRIX and Gavin06SPOKE are matrix and spoke model interpretations, respectively, of [104]. The matrix model of interpreting pull-down studies connects all prey proteins that were pulled out with a bait, while the spoke model connects only the preys with the bait. We focus on yeast PPINs, since yeast is a well-annotated organism with Gene Ontology terms. The Krogan06 and Gavin06SPOKE yeast PPINs have low overlap. To evaluate the success of our approach, we employ known complexes from the MIPS database [105,106]. We evaluate whether known MIPS complexes could be predicted using triangles and theme motifs, consisting of PPINs combined with complementary data such as SDDIs. For illustratory purposes, we use three manually curated networks of myosin-actin involvement in different cellular processes [see Additional files 1, 2, 3, and 4]

### Low overlaps of PPINs with complexes

The biological motivation for our work includes low overlap of high-throughput PPINs with known complexes. We compared the overlaps of two high-throughput PPINs, the Gavin06MATRIX and Krogan06 networks, with the MIPS protein complexes dataset. Table 1 shows full results for the overlaps of Gavin06MATRIX and Krogan06 networks to the MIPS complexes. For protein pairs that appear in both PPINs and complexes, we evaluated the number of overlapping edges PPIN ∩ *complexes*. We found *Gavin*06 ∩ MIPS has 305 overlapping edges, *Krogan*06 ∩ MIPS has 359 overlapping edges.

**Table 1:** Overlap of high-throughput PPI networks (Gavin06MATRIX and Krogan06) with the MIPS network (without triangles).

**Network**	**Edge overlap with MIPS**^***a***^	**Edges in network but not in MIPS**^***b***^
Gavin06MATRIX	305	3989
Krogan06	359	2225

Symbols below denote *E*, edges; |·|, set cardinality; ∩, intersection; -, set difference.

*a *|*E*_*network *_∩ *E*_*MIPS*_|

*b *|*E*_*network *_- *E*_*MIPS*_|

Only those edges were considered where both proteins were present in the PPI network and in MIPS.

**Figure 3:**
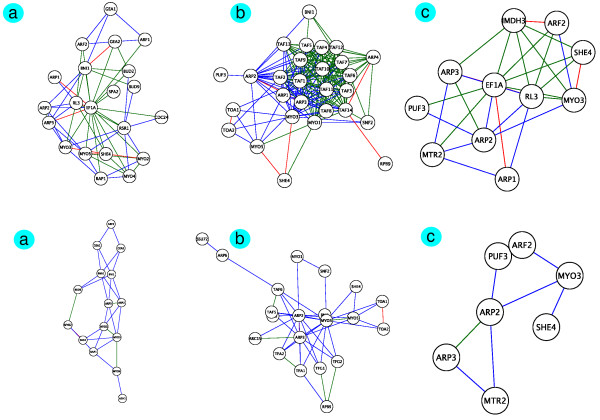
**Overlaps of our manually curated myosin-actin networks with high-throughput Gavin06MATRIX (*top*) and Krogan06 (*bottom*) PPINs**. The overlap is low. Each row shows the myosin-actin involvement in: *a*. Cytoskeleton organisation, *b*. Nucleus transcription, and *c*. mRNA translocation. Red is both PPINs and myosin-actin; blue is just myosin-actin; green is just PPIN.

Gavin06MATRIX and Krogan06 had thousands of edges connecting these same proteins, which were not in MIPS. Figure 3 illustrates the overlaps of Gavin06MATRIX and Krogan06 to manually curated myosin-actin networks; the high-throughput PPINs detected disconnected components and individual modules, but not the entire connected myosin-actin processes.

### PPI-SDDI-PPI triangles predict complexes

Given the many false negatives (missing interactions) and false positives (wrong interactions) in protein-protein interaction networks (PPINs) derived from high-throughput experiments, we evaluated the success of triangle network motifs and themes in finding known MIPS complexes. With structural domain-domain interactions (SDDIs) representing binding evidence on proteins, PPI-SDDI-PPI triangle network motifs are likely to reflect true complexes. To evaluate this, we examined the overlap of triangles from Gavin06 and Krogan06 with MIPS complexes. For the common proteins we evaluated the interactions that are true positives (overlap) or false positives (no overlap) with MIPS.

The first row of table 2 shows the low overlap between PPIN second-level neighbors (without complementary data) and MIPS complexes; where all three proteins in an indirect relation occur in MIPS complexes (denominator), rarely both PPIs occur (numerator). Despite the observed functional similarity of second-level neighbors in PPINs [62-70], second-level neighbors have overlap with MIPS lower than 1%. The other rows show that integrating complementary datatypes (CD) in a PPIN to form PPI-CD-PPI triangle network motifs results in a higher overlap with MIPS complexes. In Table 2 the second row shows the PPI-SDDI-PPI triangle overlap with MIPS complexes as a true positive rate as high as 31%; the other triangle interactions are likely false positives. For Gavin06MATRIX the triangle true positive rate is lower than for Krogan06, since Gavin06MATRIX reflects the matrix model interpretation, which resulted in 93, 881 edges including many false positives. Gavin06MATRIX has many errors when overlayed with the MIPS complex dataset. The success rate is higher for Gavin06SPOKE, since there are fewer false positives than Gavin06MATRIX.

**Table 2:** Success of triangle network motifs and themes in predicting known MIPS complexes.

**Complementary datatype**	**Gavin06MATRIX**	**Gavin06SPOKE**	**Krogan06**
None ^*a*^	936/166241 = 0.6%	516/10791 = 4.8%	914/33124 = 2.8%
SDDI ^*b*^	254/2832 = 9.0%	143/521 = 27.4%	254/1182 = 21.5%
Literature co-occurrence ^*c*^	710/5592 = 12.7%	416/1340 = 31%	502/1876 = 26.8%
Domain co-occurrence ^*d*^	2004/21876 = 9.2%	892/4268 = 20.9%	1250/4776 = 26.2%

Union of all above	2477/26468 = 9.4%	1446/6129 = 23.6%	1647/6489 = 25.4%

*a *Second-level indirect relations only

*b *Structural domain-domain interaction

*c *PubMed literature co-occurrence of protein mentions

*d *Pfam domain co-occurrence in IntAct PPIs

Fractions denote *True Positive PPIs*/*All triangle PPIs *for triangles or second-level neighbors where all three proteins occur in MIPS complexes. Triangle success in MIPS complex prediction is shown as the triangle edges that overlap with complexes. We consistently notice a lower success rate for Gavin06MATRIX than Gavin06SPOKE, which is explained by the higher number of errors in Gavin06MATRIX.

Table 3 shows that with varying confidence thresholds for SDDIs, the true positive rate changes. This shows that it is preferable to use the highest-confidence SDDIs. It also shows the significance of using SDDIs for complex prediction.

**Table 3:** PPIN triangle success in MIPS complex prediction.

**CD = structural SDDI, protein-SCOP domain assignments > confidence threshold**			
**Confid.thres.**	**Gavin06MATRIX**	**Gavin06SPOKE**	**Krogan06**

0	254/2832	143/521	254/1182
40	160/2053	91/367	168/939
50	70/1192	42/215	99/679
60	44/786	29/152	60/467
70	38/704	23/146	55/418
80	36/639	21/130	40/337
90	35/601	21/124	39/306

**CD = threading, protein-SCOP domain assignments > confidence threshold**			

**Confid.thres.**	**Gavin06MATRIX**	**Gavin06SPOKE**	**Krogan06**

medium	0/24	0/1	1/12
high	56/296	30/69	68/112
certain	205/4290	123/416	219/1099

Fractions denote *True Positive PPIs */*All triangle PPIs*. In triangles where the complementary data (CD) are SDDI structural information or threading, SCOP domain families first need to be assigned to proteins based on confidence. The confidences for protein-SCOP domain assignments are derived, for structural SDDIs based on BLAST sequence alignment similarity, and for threading they are provided by the GTD threading database. Considering different confidence thresholds for protein-SCOP domain assignments affects the MIPS complex prediction success rate.

### Triangles with other complementary data

We added to PPINs other complementary datatypes, besides structural SDDIs, to form triangles: PubMed literature co-occurrences of protein mentions, and Interpro Pfam domain co-occurrences in PPIs [107] (see methods section). Table 2 rows 3–4 show the MIPS complex overlaps with triangle network motifs using other complementary datatypes to form triangles. The triangles with other complementary datatypes exhibit little difference in their overlap with MIPS complexes. In the last row 5 where all datatypes are combined, the overlap with MIPS increases. Triangles that include SDDIs or other complementary data to match second-level neighbors have higher overlap with MIPS complexes than second-level neighbors without any complementary data. These results point to the direction of complementing the PPINs with other datatypes as triangle network motifs, rather than simple edges, for improved prediction of MIPS complexes.

Table 4 shows the individual ability of various datatypes to predict the MIPS complexes, showing the edge overlap without forming triangles. As shown under the column "Edges in edge overlap", all datatypes have moderate edge overlap with MIPS. The individual datatypes have little difference in their ability to predict MIPS.

**Table 4:** Individual ability of various datatypes to predict MIPS complexes.

Network	Number of nodes ^*a*^	Number of edges ^*b*^	Node overlap with MIPS ^*c*^	MIPS nodes not in network ^*d*^	Network nodes not in MIPS ^*e*^	Nodes in edge overlap ^*f*^	Edges in edge overlap ^*g*^	MIPS nodes not in edge overlap ^*h*^	MIPS edges not in network ^*i*^	Network edges not in MIPS ^*j*^
Gavin06MATRIX	2551	93881	584	791	1967	554	305	821	247	3989
Gavin06SPOKE	2551	22452	584	791	1967	535	232	840	320	950
Krogan06	3670	14291	1031	344	2639	994	359	381	928	2225
SDDI ^*k*^	1551	42222	515	860	1036	500	182	875	207	7375
Threading	4019	95935	1037	338	2982	1017	219	358	1129	11938
Literature co-occurrence ^*l*^	96379	170638	1056	319	95323	1235	491	140	1299	2129
Domain co-occurrence ^*m*^	3560	158704	1042	333	2518	1038	287	337	940	6064

All above combined	100443	504242	1351	24	99092	1358	979	17	1048	24156

Symbols below denote *N*, nodes; *E*, edges; |·|, set cardinality; ∩, intersection; -, set difference; ×, cross product

*a *|*N*_*network*_|

*b *|*E*_*network*_|

*c *|*N*_*MIPS *_∩ *N*_*network*_|

*d *|*N*_*MIPS *_- *N*_*network*_|

*e *|*N*_*network *_- *N*_*MIPS*_|

*f *|Nodes in *E*_*MIPS *_∩ *E*_*network*_|

*g *|*E*_*MIPS *_∩ *E*_*network*_|

*h *|*N*_*MIPS *_- Nodes in *E*_*MIPS *_∩ *E*_*network*_|

*i *|(*E*_*MIPS *_∩ (*N*_*network *_× *N*_*network*_)) - *E*_*network*_|

*j *|(*E*_*network *_∩ (*N*_*MIPS *_× *N*_*MIPS*_)) - *E*_*MIPS*_|

*k *Structural domain-domain interactions

*l *PubMed literature co-occurrences of protein mentions

*m *Pfam domain co-occurrences in IntAct PPIs

This table shows the MIPS overlaps with other network datasets (shown in the first column), indicating the ability of the various networks to predict MIPS. The MIPS network has number of protein nodes |*N*_*MIPS*_| = 1375 and number of edges |*E*_*MIPS*_| = 2099.

### Example: reconstructing distinct myosin-actin biopathways via themes of PPI-SDDI-PPI triangle network motifs

**Figure 4:**
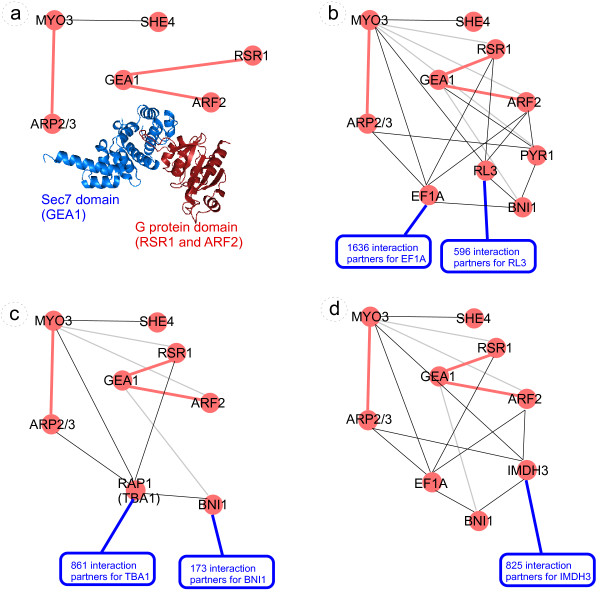
**Triangle network motifs and themes from Gavin06MATRIX**. Red lines are SDDIs and black lines are PPIs; the SDDIs did not overlap with PPIs from Gavin06MATRIX. The blue boxes show the high number of interaction partners for various proteins in Gavin06MATRIX, supporting that integration with SDDIs can help to find protein complexes. The light gray lines show additional protein connections in the dataset, which resulted in triangles. Each subfigure shows a subnetwork of the original dataset with a specific story. The subfigures represent: *a*. Myosin-actin interactions, mostly SDDIs, which occur in various processes and locations. The blue structure shown is the Sec7 domain (SCOP code a.118.3.1), which was assigned to GEA1. The red structure is the G protein domain (SCOP code c.37.1.8), which was assigned to RSR1 and ARF2. The PDB file that displays these domains as interacting, and from which the image was generated, has the code 1RE0. *b*. These SDDIs are extended with additional PPIs from Gavin06MATRIX, showing specific myosin-actin involvement in cytoskeleton organisation, *c*. nucleus transcription, and *d*. mRNA translocation.

Type I myosin motor proteins (MYO3 or MYO5) have distinct but overlapping functions in multiple cellular processes and locations [108]. Figure 4 shows examples of myosin involvements as PPI-SDDI-PPI triangle network motifs and themes derived from Gavin06MATRIX [104]. Figure 4a shows several core myosin-actin SDDIs that are common to different processes and locations. The SDDIs were validated with the structural interaction network given in [109]. For instance, Myosin type I (MYO3) has SDDIs with the ARP2/3 complex, which plays a major role in the regulation of the actin cytoskeleton, but also plays a role in actin-filament formation during transcription in the nucleus [108]. Figures 4b,c,d extend these core myosin-actin SDDIs with PPIs that are specific to different processes and locations: cytoskeletal actin organization, nuclear transcription, and asymmetric mRNA localization [110].

MYO3 is one of two type I myosins, which utilize the cytoskeleton for movement, moving along microfilaments through interaction with actin. Deletion of MYO3 causes severe defects in growth and actin cytoskeleton organization [111]. Besides myosin, SHE4 is also important for the organization of the actin cytoskeleton. SHE4 is of special interest because it is involved in all of organization of the actin cytoskeleton, asymmetric mRNA localization, and endocytosis [112]. SHE4 has similar Gene Ontology annotations as myosin.

Next, we explore whether triangle network motifs and themes in Gavin06MATRIX can help reconstruct distinct myosin-actin pathways for cellular localization of biomolecules.

#### Cytoskeletal actin organization

Figure 4b illustrates the relevant triangle network motifs. Yeast cells organize their actin cytoskeleton in a highly polarized manner during vegetative growth. Myosin type I is known to play an important role in moving membranes against actin and membrane-actin interactions. Organization of the actin cytoskeleton requires SHE4. SHE4 is a protein containing a domain that binds to myosin motor domains to regulate myosin function [112].

RSR1, BNI1, GEA1 play a role in cytoskeletal actin localization [113,114]. The correct localization of RSR1 has been shown to be critical for actin cytoskeleton organization. Localization of the Ras-like GTPase RSR1 and its regulators are required for selection of a specific growth site [115]. Regulators direct the correct localization of RSR1 in various organisms. In Figure 4b, while RSR1 interacts with both MYO3 and GEA1, it also interacts with parts of their intersecting neighborhoods. Both GO term similarity and the literature suggest MYO3/GEA1 control of RSR1. The GEA1 RAS superfamily G proteins (small GTPase) has observed SDDIs with both ARF2 and RSR1. GEA1 is a Guanine nucleotide exchange factor for ADP ribosylation factors (ARFs), involved in vesicular transport between the Golgi and ER, Golgi organization, and actin cytoskeleton organization; similar to but not functionally redundant with GEA2. An active Sec7 region in GEA1, which is the probable catalytic domain for GEF activity, is important for actin cytoskeleton activity. The mechanism by which GEA1 and GEA2 stimulate actin cable formation in a BNI1-dependent manner remains to be determined [116,117].

What is of special interest in this example is the intersection of the neighborhoods of RSR1, ARF2, BNI1 comprising EF1A-RL3, which were previously observed to have a functional significance for F-actin localization [118]. In addition, BNI1 and GEA1 appear to be connected to the ARF2 complex via PYR1 intermediary. Thus, RSR1, GEA1 and BNI1 appear to be linked to one another via EF1A-RL3-PYR1, which are also common partners of ARF2. This suggests a role of EF1A-RL3-PYR1 as the regulators for the RSR1-GEA1-BNI1 complex localization in yeast cytoskeletal actin localization [119].

Overexpression of GEA1 or GEA2 was observed to bypass the requirement for profilin in actin cable formation [116]. Profilin is an actin-binding protein involved in cytoskeleton dynamics. Profilin enhances actin growth as follows: Profilin binds to monomeric actin on the plus end of the filament inducing a shape change of the actin subunit, allowing the G-actin to replace the ADP to which it is bound by ATP and form F-actin. The F-actin then forms a heterodimer which can bind to the plus end of an actin filament. In the process of binding to the actin monomers it also stereochemically inhibits addition to the minus end [120]. On the other hand, in a separate study it was observed that loss of the activity to bind EF1A-RL3 displayed an abnormal phenotype represented by dissociated localizations of F-actin, which were co-localized in wild-type cells [118]. This observation links the two studies, suggesting that the significance of EF1A-RL3 for F-actin localization may help explain why overexpression of GEA1 or GEA2 bypassed the requirement for profilin in actin cable formation.

#### Nuclear actin and myosin I required for RNA polymerase I, II, III transcription

Figure 4c illustrates the relevant triangle network motifs. The presence of actin and nuclear myosin type I (NMI) in the nucleus suggests a role for these motor proteins in nuclear functions. Both actin and nuclear myosin I (NMI) are associated with ribosomal RNA genes (rDNA) and are required for RNA polymerase I, II, III (Pol I, II, III) transcription [121-124]. Actin and NMI are present in nucleoli as a complex physically associated with RNA polymerase I. This association appears to have a functional relevance in rDNA transcription. Altogether an actin-myosin complex is present on actively transcribing ribosomal genes and, therefore, suggests a direct involvement of actin-myosin in regulating transcription [125].

TBA1/RAP1 play a role in nucleus transciption from RNA polymerase II promoter. TBA1/RAP1 is a DNA-binding protein involved in either activation or repression of transcription, depending on binding site context; it also binds telomere sequences and plays a role in telomeric position effect (silencing) and telomere structure. In Figure 4c, RAP1 is associated with MYO3/SHE4, which transport RAP1 and actin in the nucleus and the cytoplasm. While RAP1 has PPIs to RSR1, BNI1 and ARF2, literature confirms this is an indirect relationship and instead that Myosin type I translocates RAP1 in both the nucleus and cytoplasm (precisely the myosin type I GO annotation) [126,127]. The indirect interaction of RAP1 with RSR1, BNI1 and ARF2 points to the involvement of actin in transciption.

#### mRNA localization: The SHE protein complex is required for cytoplasmic transport of mRNAs in yeast

Figure 4d illustrates the relevant triangle network motifs. A key feature of eukaryotic cells is their organization into distinct compartments, each with a distinct set of proteins. It has been shown that the sorting of many cytoplasmic proteins involves mRNA localization. Cytoplasmic localization starts in the nucleus where a first set of RNA-binding factors recognize localized mRNAs [124,128]. RNA-protein complexes that are exported to the cytoplasm associate with additional factors, such as molecular motor proteins. Such motors are required to transport their cargo along cytoskeletal filaments to the target site where the mRNA is unloaded and anchored. The SHE protein complex facilitates cytoplasmic localization of ASH1 and other localized mRNAs [129].

ARF2, EF1A, IMDH3 play a role in mRNA localization for translation. ARF2 is an ADP-ribosylation factor involved in regulation of coated formation vesicles in intracellular trafficking within the Golgi [130]. In Figure 4d, ARF2 is likely to interact with subsets of the main cluster; particularly we notice an association of ARF2 with both EF1A and IMDH3:

• **EF1A**: Translation elongation factors are responsible for two main processes during protein synthesis on the ribosome [131]. EF1A (or EF-Tu) is responsible for the selection and binding of the cognate aminoacyl-tRNA to the A-site (acceptor site) of the ribosome. EF2 (or EF-G) is responsible for the translocation of the peptidyl-tRNA from the A-site to the P-site (peptidyl-tRNA site) of the ribosome, thereby freeing the A-site for the next aminoacyl-tRNA to bind. Elongation factors are responsible for achieving accuracy of translation and both EF1A and EF2 are remarkably conserved throughout evolution (InterPro annotation).

• **IMDH3**: Involved in the amino acid biosynthesis pathway.

### Biological interpretation of PPI-SDDI-PPI triangles: A structural basis for functional similarity of second-level neighbors in PPINs

**Figure 5:**
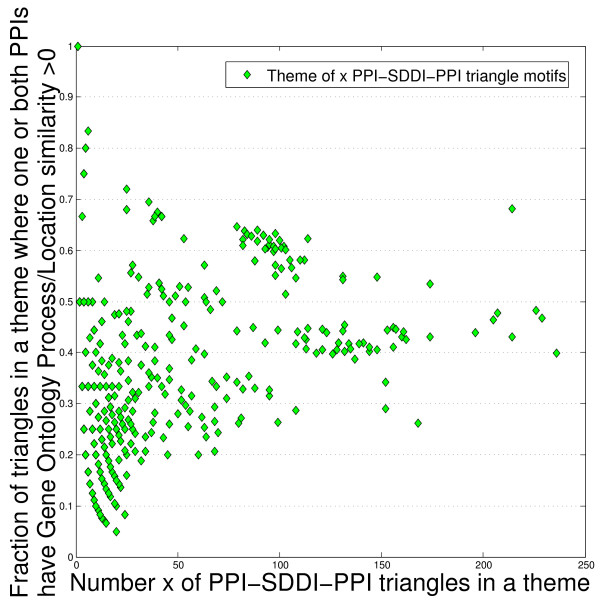
**The *x *axis is the number of triangle network motifs in themes of Gavin06MATRIX**. The *y *axis is the percentage of those triangles that have non-zero Gene Ontology similarity in their PPI edges. In many triangle network motifs both PPI edges have Gene Ontology annotation similarity equal to zero for the proteins involved.

In this section we propose an explanation for the observation that SDDIs can complement high-error PPINs to improve the finding of complexes. A structural SDDI between two proteins implies that they are likely to be observed with common groups of interaction partners in an experimental study. This especially holds in affinity purification experiments followed by mass spectrometry (AP/MS), since the bait-prey technologies used will cause structurally connected proteins to be detected as prey for similar bait protein(s). Of course this only holds for proteins that are detectable as prey [132]. A SDDI is the likely reason why two proteins are observed with common friends in PPINs from high-throughput AP/MS studies. Then, the SDDI's interaction partners are likely to be observed in different cellular components; Figure 5 shows that many of the SDDI-induced triangles have no common Gene Ontology annotation. Then SDDIs are a partial explanation for the functional similarity of second-level neighbors in PPINs. We propose this *couple-with-common-friends *model as the biological basis for finding complexes via PPI-SDDI-PPI triangle network motifs and themes; subsequently, SDDI edges in triangles can be replaced by other complementary datatypes.

#### Gene Ontology (GO) similarity in triangle PPI edges

**Figure 6:**
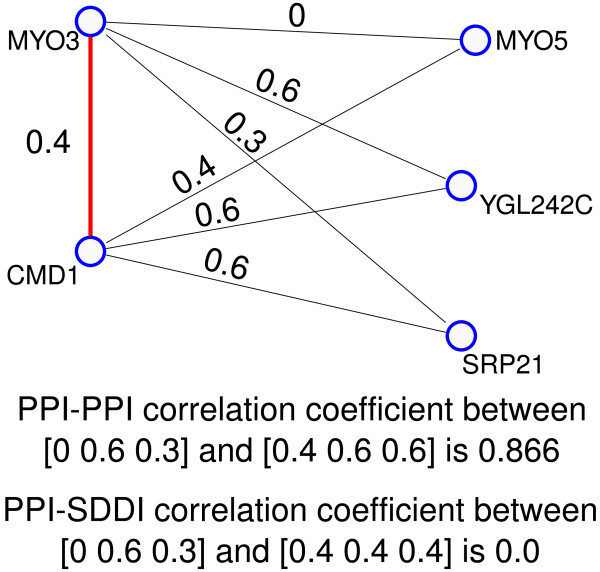
**An example of a theme from Krogan06 and the Gene Ontology similarities involved**. As shown, computing the correlation coefficients between PPI-PPI vs. DDI-PPI edges gives different correlation values.

Figure 6 shows an example of a theme from Krogan06, the GO similarities involved, and the evaluated correlations of GO similarities for the PPIs and SDDIs. Table 5 shows that in Gavin06MATRIX and Krogan06 triangle network motifs, SDDIs have significantly higher GO similarities than protein-protein interactions (PPIs). Evaluation of GO similarities in the PPI-SDDI-PPI triangles in Gavin06MATRIX and Krogan06 shows that the PPIs in a triangle represent similar functions and process/location involvements [133]. Table 5 shows a correlation analysis confirming that the PPI-PPI GO similarities (function, process, location) are higher correlated than the SDDI-PPI GO similarities. The correlation of GO similarities of PPI edges in a triangle implies that the SDDI brings together two PPIs involved in similar functions and processes/locations. Figure 5 shows that some triangles' PPIs have GO similarity of zero, hinting at errors. This may also show some promise for finding errors based on GO similarity.

**Table 5:** Gavin06MATRIX PPI-SDDI-PPI triangles and Krogan06 PPI-SDDI-PPI triangles: Gene Ontology (GO) similarities and correlations.

	**Correlation** **Gavin06MATRIX**	**Correlation** **Krogan06**
**GO Functional similarity**		

Average over protein pairs in SDDI edges	0.48	0.37
Average over protein pairs in PPI edges	0.18 and 0.18	0.35 and 0.36
PPI-PPI similarity correlation coefficient	0.88	0.76
SDDI-PPI similarity correlation coefficient	0.16 and 0.18	0.58 and 0.59

**GO Process/Location similarity**		

Average over protein pairs in SDDI edges	0.71	0.67
Average over protein pairs in PPI edges	0.28 and 0.27	0.67 and 0.67
PPI-PPI similarity correlation coefficient	0.97	0.6
SDDI-PPI similarity correlation coefficient	0.08 and 0.09	0.46 and 0.45

Often, the two PPIs of a triangle have close GO functional/process/location similarity.

#### Why are few SDDIs detected in high-throughput PPINs experiments?

**Figure 7:**
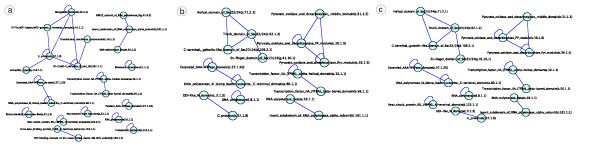
**SDDIs that were detected as PPIs in PPINs: (*a*) Krogan06, (*b*) Gavin06SPOKE, (*c*) Gavin06MATRIX**. Only one more SDDI was detected in Gavin06MATRIX than in Gavin06SPOKE, pointing to the high number of FPs in the matrix model.

Table 6 shows that few SDDIs overlap with PPINs, even when considering the highest-confidence SDDIs only. Figure 7 shows a visualization of the SDDIs overlapping with PPINs. The visualization shows that most of these SDDIs are part of *highly connected *components. To assess whether the size of the connected SDDI components that overlap with PPINs is significant, we compared the connected SDDI components to randomly selected SDDIs from the SCOPPI database [134]. We performed 1,000 trials of randomly picking 100 SDDIs from SCOPPI, and we examined how many of these SDDIs were connected each time; on average only 8 SDDIs were connected, a size much smaller than the connected SDDI components that are shown in Figure 7. These results highlight the significance of complementing PPINs via PPI-SDDI-PPI triangles.

**Table 6:** Few SDDIs overlap with PPINs derived from high-throughput experiments and MIPS complexes.

	SDDI	Gavin06-MATRIX	Gavin06- SPOKE	Krogan06
SDDIs total with both proteins in MIPS and PPIN	SCOPPI ^*a*^	71	71	238
	Threading ^*b*^	3404	3404	9615
SDDIs supported by PPIs in both MIPS and PPIN	SCOPPI	14	9	25
	Threading	61	56	99
SDDIs supported by PPIs in MIPS but not PPIN	SCOPPI	0	5	5
	Threading	20	25	107
SDDIs supported by PPIs in PPIN but not MIPS	SCOPPI	37	30	48
	Threading	144	72	131
SDDIs supported by PPIs in neither PPIN nor MIPS	SCOPPI	20	27	160
	Threading	3179	3251	9278

*a *protein-SCOP > 90 conf.

*b *protein-SCOP CERTAIN conf.

## Conclusion

### How many SDDIs are needed to predict all complexes for an entire PPIN?

**Figure 8:**
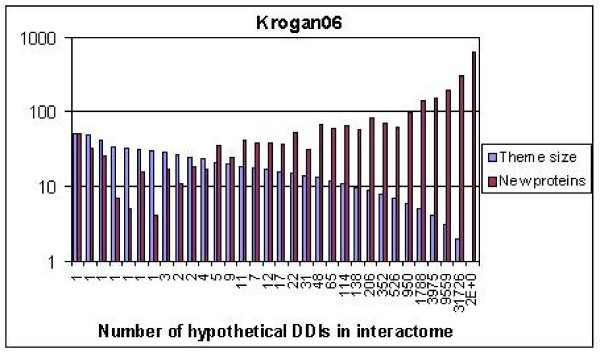
**Starting from the few SDDIs (*x*-axis) with the largest theme sizes (blue), and progressively moving to the many SDDIs with the smallest theme sizes**. This allows one to eventually find the complexes for all proteins in the PPIN (red).

Figure 8 is an attempt to predict how many structural SDDIs would be needed for triangles to predict the true positives involving all proteins in a typical PPIN, such as Krogan06. We took all second-level indirect neighbors found in the Krogan06 interactome and, where there was no PPI, added a "hypothetical" SDDI to form PPI-SDDI-PPI triangles. For each SDDI we calculated its theme size, i.e., how many pairs of PPIs the SDDI connected into triangles. Then, we took the theme sizes in decreasing order from 100 to 1, as shown by blue bars in Figure 8. For each theme size, we indicate on the x-axis how many SDDIs had that theme size, and the red bar shows how many newly encountered proteins were included for that theme size. As the x-axis shows, one could start by finding true positives for the few SDDIs with the largest themes, progressively moving to the many SDDIs with the smallest themes. Somewhere in the middle of the *x*-axis, one would have predicted the true positives for about half of the proteins in the PPIN. However, one would still need to use many SDDIs with the smallest themes, to find all true positives in the Krogan06 PPIN. Therefore, although about half of the true positives could be found with no more than 100 SDDIs, one would need significantly more SDDIs to find all true positives involving all proteins.

SDDIs and the PubMed co-occurrences relate to two different aspects. SDDIs are based on experimental results that are likely to imply a structural interaction. In the case of SDDIs, we can use all information found by mapping structural domains to proteins using BLAST sequence similarity and still get good prediction accuracy. On the other hand, for literature we have to apply a strict filtering, keeping only the top 1% of protein co-occurrences appearing in PubMed as complementary data. We observed that the literature co-occurrences appear to give slightly better results than using SDDIs as complementary data. The main limitation of SDDIs at present is the sparsity of known structural interactions. Since PubMed is expected to grow faster than structural knowledge, using literature co-occurrences might give even better prediction accuracy in the future, as long as a strict cut-off is set.

## Conclusion

With the amount of PPINs from high-throughput experiments, structural data and literature-based interactions on the rise, we studied their combined ability to predict known complexes. We found a low overlap of PPINs derived from high-throughput studies with known complexes, as well as low overlap with structural domain-domain interactions.

We proposed PPI-SDDI-PPI triangle network motifs as a model for analysing PPINs and predicting complexes. PPI-SDDI-PPI triangles have higher overlap with MIPS complexes than random second-level neighbors, indicating that structural SDDIs are useful for complementing PPINs in triangles to create a more complete picture of protein cellular involvement. We complemented PPINs with several other datatypes besides SDDIs to create triangle and theme motifs, resulting in similar overlaps with complexes. Themes of PPI-SDDI-PPI triangles helped us to reconstruct complexes in myosin-actin processes that were not detected by PPINs. Our approach is useful for finding true positives in PPINs, as structural knowledge on proteins increases in the future.

SDDIs partially explain the high functional similarity of second-level neighbors in PPINs. A SDDI may cause a structurally connected pair of proteins to be observed with common interaction partners in high-throughput affinity purification experiments followed by mass spectrometry (AP/MS) that use bait-prey technologies. We examined why some SDDIs are detected in PPINs, and we found that SDDIs detected by PPINs are part of highly connected components/complexes, therefore they are more likely to be detected by experimental studies.

## Methods

In this section we give an overview of the methods used in this study. Figure 2 illustrates the overall workflow of the process.

### PPI-CD-PPI triangle network motifs

PPI-CD-PPI triangles contain three proteins connected by two PPIs and an edge of a complementary datatype (CD), such as a structural SDDI; in this case, we refer to PPI-SDDI-PPI triangles, as Figure 1a shows. Our method can be viewed as finding bicliques in a PPIN, and then connecting second level neighbors via *complementary datatype edges*. For extracting second level neighbors in large networks we used the HIERDENC algorithm, described in [62,135]. Figure 1b and 1c show that PPI-CD-PPI triangles imply that an experiment detected PPIs *A *↔ *B *and *B *↔ *C*, while a CD edge *A *↔ *C *exists, such as a structural SDDI. In a PPIN second level neighbors (a pair of PPIs) may be involved across cellular space and time in different processes and locations. Connecting second level neighbors to each other via CD edges gives confidence that the second level neighbors interact at the same cellular space and time [136-138]. Triangles likely represent a protein complex [139,140].

Let *σ*_*SDDI *_denote the number of PPI-SDDI-PPI triangles a structural SDDI is involved in. A structural SDDI may be involved in *σ *≥ 1 triangles, which we refer to as a *theme*. A theme is given by the *σ *common interaction partners (intersecting neighborhoods) of a SDDI's protein pair, and some PPIs in a theme may be False Positives.

### Complementary datatypes

As structural information to complement PPINs, we used the SCOPPI database, which contains SDDIs observed in known protein complex structures [134]. To assign domains, we BLASTed the sequences of all proteins in the "Saccharomyces Genome Database" (which includes yeast PPINs) against all domains sequences of SCOPPI. We considered only BLAST hits with an E-value ≤ 0.01 and a sequence identity percentage *s *≥ 30%. In addition, we required 75% of the domain to appear in the protein.

Other complementary datatypes (CD) edges we used included The Genomic Threading Database (GTD) [141]. GTD contains yeast protein assignments to SCOP domain structural annotations and interacting structures. An assigned Confidence value gives an indication of the strength of a hit, ranging from "certain" to "guess", which is based on a P-value measure of significance.

The next CD dataset we used was PubMed literature co-occurrences of protein mentions. To extract these, we used the GoPubMed protein mention extraction algorithm to assign proteins to all PubMed documents [142]. Then, we used a version of the Blosum co-occurrence score to find if two proteins *p*_1 _and *p*_2 _co-occur frequently in PubMed documents: . A cutoff of 10 was strict enough to filter out the majority of protein co-occurrences in PubMed, resulting in a network of 170,638 edges. The last CD dataset we used was Interpro Pfam domain co-occurrences in PPIs. For this, we took all IntAct yeast PPIs and assigned to the proteins Pfam domains from InterPro [107]. Then, we used the Blosum co-occurrence score to find which Pfam domains co-occur frequently in the IntAct yeast PPIs. Based on the most co-occurring Pfam domains, we build a network over the yeast PPIs.

### High-throughput PPINs and known complexes

We use two yeast PPINs that we denote as Gavin06 [104] and Krogan06 [103]. For Gavin06 we used both the matrix and the spoke model to interpret it, which we refer to as Gavin06MATRIX and Gavin06SPOKE throughout the text. Gavin06MATRIX had 93,881 edges, while Gavin06SPOKE had 22,452 edges. Krogan06 had 14,292 edges, consisting of the binary interactions as provided by the publication. For validation, we used MIPS complexes [105,106]. For MIPS we used the SPOKE model for the interpretation of complexes, since otherwise the result would be biased to give a high overlap with the PPINs [see Additional files 5, 6]. The MIPS complexes had 2,099 edges.

Moreover, for our illustrations we manually curated three network examples from the literature, representing myosin-actin involvement in cytoskeleton organisation, nucleus transcription, and mRNA translocation. Developing these networks involved reading papers from the biomedical literature and recording any interaction(s) described in the articles.

### Gene Ontology similarity

It is likely that a PPI is not physical, but a false positive, which may be detected by a GO similarity of zero. PPIs with a GO similarity of zero hint at false positives. For calculating the similarity based on Gene Ontology terms, we searched for GO terms in the current abstract and compared them to the set of GO terms assigned to each gene candidate. For each potential tuple taken from the two sets (text and gene annotation), we calculated a distance of the terms in the ontology tree. These distances yielded a similarity measure for two terms, even if they did not belong to the same sub-branch or were immediate parents/children of each other. The distance took into account the shortest path via the lowest common ancestors, as well as the depth of this lowest common ancestor in the overall hierarchy (comparable to Schlicker et al., 2006 [133]). The distances for the closest terms from each set then defined a similarity between the gene and the text [142].

### Correlation

We computed the correlation coefficient between *A *and *B*, where *A *and *B *are matrices or vectors of the same size. A matrix entry contains a measure of Gene Ontology similarity (0 – 1) for a protein pair involved in a PPI or SDDI. We used the matlab corr2 correlation coefficient:



### HIERDENC supplementary material

We implemented the HIERDENC online database, which contains all of the datasets we used. HIERDENC helps a user to visualize and find true positives in PPINs via triangles of high-throughput PPINs and complementary data.  or 

## Authors' contributions

All authors read and approved the final manuscript. BA planned the paper, carried out most of the experiments, wrote the software, wrote the python scripts for making the networks, built the manually curated networks, and wrote most of the paper. CW helped in conceptualising the paper with discussions and provided the complementary data. DL helped in formulating the paper with discussions. MS supervised the work and contributed discussions and ideas.

## Supplementary Material

Additional file 1 **An excel file with Uniprot accession numbers for all protein names used in the text.** Click here for file

Additional file 2 **Manually curated network example from the literature, representing myosin-actin involvement in cytoskeleton organisation.** Click here for file

Additional file 3 **Manually curated network example from the literature, representing myosin-actin involvement in mRNA translocation.** Click here for file

Additional file 4 **Manually curated network example from the literature, representing myosin-actin involvement in nucleus transcription.** Click here for file

Additional file 5 **MIPS complexes dataset.** Click here for file

Additional file 6 **A script for converting MIPS complexes to a SPOKE model network.** Click here for file
